# Pig feeds rich in rapeseed products and organic selenium increased omega-3 fatty acids and selenium in pork meat and backfat

**DOI:** 10.1002/fsn3.182

**Published:** 2014-12-03

**Authors:** Eli Gjerlaug-Enger, Anna Haug, Mari Gaarder, Kari Ljøkjel, Ragna Sveipe Stenseth, Kerstin Sigfridson, Bjørg Egelandsdal, Kristin Saarem, Per Berg

**Affiliations:** 1 NorsvinFurnesveien 223, 2319, Hamar, Norway; 2 Department of Animal and Aquacultural Sciences, Norwegian University of Life Sciences1432, Ås, Norway; 3 Nortura SAPostboks 360 Økern, 0513, Oslo, Norway; 4 Felleskjøpet FôrutviklingNedre Ila 20, 7018, Trondheim, Norway; 5 Felleskjøpet Agri StangeSilovegen 6, 2335, Stange, Norway; 6 Lantmännen CerealiaNesttunveien 90, 5221, Nesttun, Sweden; 7 Department of Chemistry, Biotechnology and Food Science, Norwegian University of Life Sciences1432, Ås, Norway

**Keywords:** Meat, meat and fat quality, omega-3 and omega-6 fatty acids, pig, selenium, slaughter pig efficiency

## Abstract

The concentration of omega-3 fatty acids and selenium (Se) is generally too low in the Western diet. But as the nutrient composition of pork meat and adipose tissue is influenced by the feed given to the animals, the product can be changed to support nutrient demands. Half (297/594) the pigs were given a feed concentrate based on low-glucosinolate rapeseed products (RS), while the other half was fed a traditional concentrate (Contr): The RS feed had an omega-6/omega-3 ratio of 3.6:1, and the Contr feed had a ratio of 8.9:1, and both feeds were supplemented with 0.4 mg Se/kg (organic Se: inorganic Se, 1:1). There was a small difference in growth rate, but no differences in feed conversion ratio, lean meat percentage, carcass value, and margin per pig for the two groups. There were no differences in meat quality between the two groups, but there were differences in technological fat quality. The RS pigs contained about 2 times more alpha-linolenic acid in the backfat and 41% more in the meat (M. longissimus dorsi) compared to the controls. The concentration of EPA, DPA, and DHA were 42% and 20% higher in backfat and meat of the RS pigs compared to the control pigs respectively. The ratio between omega-6/omega-3 fatty acids were 4.7 in the meat and 4.0 in the backfat in the RS pigs, and the corresponding values were 6.6 and 8.0 in the control pigs. The selenium content was 0.3 mg/kg meat in both groups. The study showed that a portion of the present pig meat (175 g) provided the daily recommended intake of Se for men and women and about 1/6 of proposed reference intake of omega-3 LCPUFA (250 mg/day) to reduce the risk of CVD thereby providing a meat that is somewhat healthier for the consumer.

## Introduction

High quality meat with a healthy nutrient composition is in demand. Pork meat is an appreciated, all-purpose lean meat, and the consumption is high (Kjøttets tilstand 2013). Pork meat is rich in protein and other nutrients, and many choose meat instead of fish, despite the consumers' knowledge that fish contains more omega-3 fatty acids and selenium (Se). The concentration of Se in pork meat in Scandinavia is approximately 0.1 mg/kg (DTU 2009; Matportalen 2014) while fish fillets contain about 3 to 4 times more (DTU 2009; Matportalen 2014). In pork meat, the amount of the very long-chain omega-3 (n3) fatty acids eicosapentaenoic acid (EPA, 20:5n3), docosapentaenoic acid (DPA, 22:5n3), and docosahexaenoic acid (DHA, 22:6n3) is reported to be approximately 0.4 g/kg (DTU). In cod, it is 2.6 g/kg, while in fatty fish such as salmon it is approximately 28 g/kg (DTU), showing that fish fillet is a much better source for omega-3 fatty acids than pork meat.

Omega-3 fatty acids have been shown to prevent lifestyle diseases such as cardio-metabolic and inflammatory disorders (Simopoulos 2002; Mozaffarian 2008; Galli and Calder 2009). The amounts of omega-6 (n6) and omega-3 fatty acids and the ratio between these fatty acids are unbalanced in Western society (Simopoulos 2002). The omega-3 and omega-6 PUFAs compete in binding to enzymes and incorporation into cells and cell membranes, where they exert effects on cellular metabolism. Arachidonic acid (AA, 20:4n6) and EPA are converted to different eicosanoids that may have opposing effects in the development of noncommunicable diseases, such as atherosclerosis, cancer, inflammation, and insulin resistance (Simopoulos 2002; Schmitz and Ecker 2008). In addition, the ratio between omega-6 and omega-3 fatty acids influence several processes at the cellular level including cell growth, multiplication, apoptosis, and cell survival (Schmitz and Ecker 2008). An increase in the ratio of AA to EPA may increase the risk of thrombosis, but a reduction in the ratio AA/EPA will have the opposite effect, as is clearly seen among the inuits in Greenland when eating their traditional diet. Each meal triggers an inflammatory response, and the ratio between omega-6 and omega-3 fatty acids is an important determinant of the magnitude of the postprandial inflammatory response (Margioris 2009).

Arachidonic acid has a high binding capacity to enzymes such as COX (Christophersen and Haug 2011), and endocannabinoids derived from AA have been shown to play a role in adiposity (Alvheim et al. 2012), which may be another reason to avoid high intakes of AA in the diet. Arachidonic acid is only present in animal tissue, and some diets recommend eating high fat foods such as, for example, bacon and lard, claiming that this is good for you. In this setting, a reduction of AA might be advantageous. Pig feed supplemented with fish oils rich in EPA and DHA may give fish taste of the meat product (Øverland et al. 1996; Bryhni et al. 2002). However, when adding the omega-3 fatty acid ALA from vegetable sources to the feed, the animal itself is able to convert some of ALA to EPA, DPA, and DHA, and the meat product will not have the problems with the fish taste (Tikk et al. 2007; Beaulieu et al. 2009).

Selenium supplementation to the feed increases the content of muscle Se (Surai 2006). Moreover, Se is incorporated into selenoproteins, which have a wide range of positive effects on health such as antioxidative and anti-inflammatory effects, while an additional Se intake may benefit people with a low Se status (Rayman 2012). The concentration of Se in wheat grown in Norway is low (wheat grown at the Norwegian University of Life Sciences in Ås, Norway contains less than 20 mg of Se/kg wheat (own results). The commercial pig feed concentrate is therefore supplemented with Se to avoid deficiency in the animals, and the supplementation is given in the form of inorganic Se (sodium selenite). There are benefits from the Se supplementation to pigs: improved product quality for human consumption, with an increased Se concentration and decreased drip loss during meat storage (Surai 2006). However, much of the sodium selenite added to the feed concentrate is excreted, and not incorporated into the meat. The supplementation of the feed with Se-enriched yeast or selenomethionine is much more efficient if the intention is to increase the Se concentration in meat for the benefit of the human consumer (Payne and Southern 2005; Fisinin et al. 2008).

Commercial swine feed is cereal- and soybean meal-based, giving about 10 times higher amount of omega-6 compared to omega-3 fatty acids. The diet eaten by pigs in their natural habitats (wild environments) consists of seeds, plants, insects, etc., providing many minerals, micronutrients and plant antioxidants, and since the main fatty acid in grass and green plants is ALA, this feed and has a much higher proportion of omega-3 fatty acids compared to omega-6 fatty acids. Nutrient components in meat, such as Se and long-chain fatty acids, closely depend on the content of these nutrients in the feed given to the animals. It has been shown that adding omega-3 fatty acids from rapeseed products (RS, *α*-linolenic acid, ALA, 18:3n3) to pig feed increased the concentrations of ALA, EPA, DPA, and DHA in pig meat, and improved the ratio between omega-6 and omega-3 fatty acids (Bertol et al. 2013). Further, dietary supplements of Se-enriched yeast increase Se concentration of the meat (Surai 2006).

We suggest that all meat available for human consumption should have a favorable low ratio between omega-6 and omega-3 fatty acids, with as much as possible of the long-chain omega 3 fatty acids (AA/EPA+DPA+DHA), as well as a concentration of selenium that goes toward the content in fish. The currently exploited sources of the long-chain omega-3 fatty acids EPA, DPA and DHA is limited because of ecological limitations on total fish production in the sea. Therefore, every step toward increasing the concentration of these fatty acids in the regular human diet from sources other than seafood is of importance. When EPA and DHA come from animal foods, rather than as purified dietary supplements, they will be ingested together with antioxidant nutrients that are important for the prevention of peroxidation in vivo (following ingestion) such as Se, glutathione (plus glutathione precursor amino acids), carnosine and taurine. These antioxidant nutrients can have important antimutagenic, anticarcinogenic, and anti-inflammatory properties, and may very likely synergize with many of the protective effects of long-chain omega-3 fatty acids. Therefore, optimizing the content of Se and omega-3 and omega-6 fatty acids in meat presents a better strategy for increasing the intake of Se and very long-chain omega-3 fatty acids to humans, rather than relying on supplements or fish resources. Further, the increased use of locally produced or short-traveled oilseed products can increase the self-sufficiency of feed for pigs in Nordic countries, thereby reducing feed import from other continents.

The objective of the present study was to provide a more healthy pig meat for human consumption, with more Se and better ratio of omega-6 and omega-3 fatty acids, by simple and practical modifications in the diet for pigs.

## Materials and Methods

### Animal care

The experimental research on animals followed internationally recognized guidelines. All animals were cared for according to the laws and regulations controlling experiments with live animals in Norway (The Animal Protection Act of 20 December 1974, and the Animal Protection Ordinance Concerning Experiments with Animals of 15 January 1996), according to the rules given by the Norwegian Animal Research Authority.

### Feeding experiment

The feeding experiment was conducted from March to June of 2012, and in total 594 pigs were included in the study. The pigs were commercial Norwegian crossbred pigs after a Norsvin Landrace × Yorkshire dam and a Norsvin Duroc terminal sire. The pigs were performance tested on ad libitum feeding regimes, with mixed sex groups of 12 pigs per pen. Pigs in every second pen was given the new diet, while the pens in between were given a commercial diet.

Pigs were individually tagged and weighted at start of the test, halfway and at the end of the test (Table1). The total feed consumed was recorded at the group level (*N* = 64), and pigs were tattooed before finishing a test for traceability at the slaughter house. Slaughtering was performed in five weekly batches at a commercial abattoir, and the carcasses were transported to a partial dissection line at Animalia, the Norwegian Meat and Poultry Research Centre. People working at this dissection line are educated and trained for research on dissection and meat- and fat quality analysis. The average carcass weight was 82.4 kg, and they were slaughtered 1 to 5 weeks after the last weighing.

**Table 1:** Numbers of animals per variable, mean, standard deviations of means (SD), minimum and maximum values for pigs fed control feed (Contr), and concentrate based on rapeseed products (RS)

Variable	Feed	*N*	Mean	SD	Minimum	Maximum
Weight start	Control	296	32.4	4.3	22	47
	RS	296	31.2	4.2	21	45
Weight middle	Control	294	61.8	7.9	43	84
	RS	295	60.1	7.3	43	83
Weight before slaughtering	Control	291	99.0	9.5	77	123
	RS	292	95.3	9.6	73	123
Slaughter weight	Control	291	83.0	4.1	63.5	98.4
	RS	292	81.8	4.5	66.8	98.0

### Experimental diets

The half of the pigs was given a concentrate containing low-glucosinolate RS, while the other half received a corresponding traditional pig feed (Contr). In traditional soya- and cereal-based concentrates for pigs, the omega-6/omega-3 ratio ranged from 8:1 to 12:1. The major feed components in the RS and Contr feeds are presented in Table2, and in the current study, the Contr feed had an omega-6/omega-3 ratio of approximately 9:1 (Table3). In the RS feed, a combination of rapeseed and rapeseed cake was used in order to target the omega-6/omega-3 ratio near 4:1. The RS contributed with a significant amount of protein to the feed, thus the protein from the soya was reduced to correspond to the increased protein content from RS. The feeds were balanced to an identical amino acid profile by using synthetic amino acids as l-lysine, dl-methionine, and l-threonine.

**Table 2:** Major feedstuff compounds (in %) in the control feed concentrate (Contr) and the feed concentrate supplemented with rapeseed products (RS) (Intact rapeseeds and rapeseed expeller)

Feedstuff compounds	Control	RS
Soybean meal	14.1	5.4
Rape expeller	0	13.0
Rapeseeds	0	5.6
Barley	59.0	50.2
Wheat	20.0	20.0
Animal fat	1.1	0

**Table 3:** Analysed values of fatty acid profile and levels of glucosinolates in the control feed concentrate (Contr) and the concentrate supplemented with rapeseed products (RS)

Fatty acids	Control	RS
C14:0	0.8	0.1
C16:0	21.1	9.0
C17:1	0.2	0.1
C18:0	8.7	1.8
C18:1c9	23.9	46.0
C18:2 n6	36.6	31.3
C18:3 n3	4.1	8.6
n6/n3	8.9	3.6
Glucosinolates total, *μ*mol/g	0	1.12
Glukonapin, *μ*mol/g	0	0.35
Progoitrin, *μ*mol/g	0	0.51
4-Hydroxyglucobrassicin, *μ*mol/g	0	0.15

The energy percentage (E%) of ALA was 0.65% in the control feed and 1.4% in the RS feed, whereas the E% of LA was 6.0% in the control feed and 5.1% in the RS feed.

The feed concentrate was given 0.4 mg of Se, in which 0.2 mg of Se was from an organic source (Sel-Plex Premix, Skansen, Trondheim, Norway), and 0.2 mg of Se was from an inorganic form (Selenpremiks, Skansen, Trondheim, Norway). The energy level was 9.24 MJ/kg, 0.79 g of apparent ileal digestible lysine/MJ, and the structure was similar for both types of concentrate. Due to the use of RS, the feed and feedstuff were analyzed for glucosinolates. Despite a high volume of RS, the concentration of glucosinolates was significantly below the recommended max level of glucosinolates of 2 mmol/g DM.

### Meat and fat quality analysis

Meat and fat quality measurements were carried out on samples from the glycolytic loin muscle M. longissimus dorsi (LD) and subcutaneous backfat on the carcass dissection day, that is four days post-mortem. The analysis of meat quality traits: drip loss (The EZ-DripLoss method), pH (WTW, pH 330i), meat color (Minolta Chroma Meter CR-400, Konica Minolta optics, Inc., Osaka, Japan; the L*, a* and b* values), as well as intramuscular fat, moisture and protein in meat (The FOSS FoodScan Tm near-infrared spectrophotometer), was preformed with the methods described by Gjerlaug-Enger et al. (2010a). The fat quality was analysed using the method known as NIR-predicted (Near Infrared Spectroscopy) moisture content in subcutaneous fat (The FOSS FoodScan Tm, FOSS, Hillerød, Denmark), which was developed by Gjerlaug-Enger et al. (2010b). Meat quality measurements were tested on 20 pigs from each group, and technological fat quality was tested on 30 pigs from each group. The ratio between castrates and females, slaughter weight and lean meat percentage was similar for the animals in these experimental groups.

### Sensory

A sensory investigation was performed to test the eating quality of sausages, neck chops, and bacon. A triangle test with trained workers was also performed at the slaughter house (Nortura, Oslo, Norway). The assessors were presented with three products, two of which were identical and the third was different. The assessors were asked to state which product they believed was the odd one (ISO 4120:2004; Sensory analysis – Methodology – Triangle test). Moreover, the experiment was run with two parallels, and the probability (*P*) for each assessor arriving at a correct response by guessing was *P* = 1/3.

### Fatty acid analyses of feed, meat, and fat

Fatty acid composition of 15 ld-muscle samples and 15 backfat samples from each group of pigs were determined by gas chromatography (GC), after lipid extraction and direct methylation according to O'Fallon et al. (2007) using 1 g of feed, fat, or meat. Subsequently, the fatty acid methyl esters were analysed with a 6890N GC, with a split/splitless injector, a 7683B automatic liquid sampler and flame ionization detection (Agilent Technologies, Palo Alto, CA). Separation was performed with a CP-SELECT CB FOR FAME (200 m × 0.25 mm i.d. × 0.25 *μ*m film thickness) fused silica capillary column (Varian Inc., SpectraLab, Ontario, Canada). The temperature program started at 70°C with 4 min hold, ramp 20°C/min to 160°C, 3°C/min to 230°C with 15 min hold. The carrier gas was H_2_, and a pressure of 309.4 kPa was used. The fatty acid analysis was performed through auto-injection of 1 *μ*L of each sample at a split ratio of 30:1, a H_2_ flow of 68.4 mL/min and a temperature of 280°C. The flame ionization detector temperature was at 290°C with H_2_, air and N_2_ make-up gas flow rates of 40, 450, and 45 mL/min respectively. The sampling frequency was 10 Hz, and run time for a single sample was 91 min. Fatty acid peak areas were corrected by theoretical response factors (Ackman and Sipos 1964), and standard fatty acids of a known composition were run to identify the fatty acids in the samples. Lastly, muscle control samples were extracted, methylated and analysed for every 10th sample.

### Determination of selenium in muscle

Selenium concentrations in the muscle of 5 pigs in each group were analysed by atomic absorption spectrometry with a hydride generator system (Norheim and Haugen 1986), using Varian SpectrAA-30, SpectraLab, Ontario, Canada with a VGA – 76 vapor generation accessory. Before analysis, each sample was prepared by oxidative digestion in a mixed solution with concentrated nitric and perchloric acids, using an automated system with a Tecator 1012 Controller and a 1016 Digester heating unit. The method is accredited (NS-EN ISO/IEC 17025), and the quality control system used regular analyses of a pork liver (GWB) with 0.94 ± 0.05 g Se g^−1^ and a bovine muscle (BCR 184) with 0.183 ± 0.012 g Se g^−1^ as reference materials. The detection limit was 0.01 g g^−1^, and the quantification limit 0.03 g g^−1^.

### Statistical analyses

Computations for slaughter pig efficiency were performed using General Linear Model in SAS (Statistical Analysis System Institute, Inc., Cary, NC). Models included as fixed effects: feed (RS/Contr.), feeding regime (dry/wet), sex, herd of birth (piglet supplier), and regression of start weight. Fatty acid composition in meat and fat were analysed by student *t*-tests in Excel.

## Results and Discussion

### Feeding experiment

In Table4, the least square means (LS-means) for slaughter pigs efficiently and the economy for the production of pigs are presented. Among the effects in the model, the feed tested (Cont vs. RS) had a smaller effect than sex, herd, and feeding-regime (wet vs. dry) for all traits tested. The pigs fed the experimental feed (RS) had both a lower growth and lower feed consumption than the control feed group. There appeared to be no differences in feed conversion rate (FCR) between the treatments. The difference in growth, at approximately 23 g/day, is equal to 2 days for a slaughter pig period, and there was no difference in the lean meat percentage for the two groups. Economical results were similar for the Contr and RS pigs, which is important in view of potential future commercial slaughter pig production with the new rapeseed-based diet.

**Table 4:** Least square means for the effect of feed in slaughter pigs. Fixed effects were feed (RS/Contr), feeding regime (dry/wet), sex, herd of birth (piglet supplier) and regression of start weight. One EUR = 8.46 NOK (February 2014)

	Control	RS	*P*
Growth, g/d	975	952	0.001
Lean meat, %	59.9	59.6	0.321
Income per animal, EUR	226	224	0.199
Feeding cost per animal, EUR	77	75	0.010
Margin, EUR	150	148	0.390

### Experimental diets

The RS feed was designed to have an omega-6/omega-3 fatty acid ratio of approximately 4:1.

The idea behind the feed composition in the current experiment was to use RS, rather than oil, to change the fatty acid composition. Intact rapeseeds and rapeseed expeller contain 42.5% and 9.5% oil respectively. We chose these feed components due to a smaller risk of oxidization of products with seeds compared with rapeseed oil. Rapeseeds and rapeseed expeller both have a physical protection of the oil, in addition to more antioxidative components that protect the unsaturated fatty acids. Another reason was the availability of the feed components. Rapeseeds and rapeseed expeller are already used at the feed mills, while an introduction of rapeseed oil makes it necessary to build new equipment in the mills. Moreover, rapeseeds and rapeseed expeller are also lower in price than rapeseed oil.

Rapeseed products also contribute with rapeseed proteins, which made it possible to reduce soybean meal in RS feed to almost one-third of the level in Contr feed in the amino acid balanced diets (Table2). The concentration of glucosinolates can be a problem with such large amounts of RS, and glucosinolates has to be analyzed and kept under control. The finishing production traits were in accordance to other studies with glucosinolates and pig performance (Bourdon and Aumaître 1990; Siljander-Rasi et al. 1996).

### Technological meat and fat quality traits

The basic statistics of the technological meat and fat quality traits studied are presented in Table5. The results indicated that the two groups tested had similar meat quality. One exception to this was the b* value of meat, in which the RS pig meat seemed to have less yellow color in the meat. In accordance with these findings, Okrouhlá et al. (2012) also found significant effects of rapeseed meal on L* value and b* value of M. longissimus dorsi, but there are also many studies without any relationship between meat color and the rapeseed feeding of pigs (Siljander-Rasi et al. 1996; Roth-Maier et al. 2004).

**Table 5:** Technological meat and fat quality measurements in meat (*M. longissimus dorsi*) and subcutaneous fat from pigs fed control feed (Contr) and concentrate based on rapeseed products (RS). Number of animals analysed were 15 × 2 and 20 × 2 for meat and fat respectively. The mean, SEM and *t*-test (*P*) in meat and in subcutaneous fat for the two treatment groups are shown

	Control		RS		
	Mean	SEM	Mean	SEM	*P*
EZ-DripLoss (%)^1^	5.4	0.3	4.7	0.3	0.140
Ultimate pH^1^	5.53	0.01	5.53	0.01	0.898
L^*^ value^1^	48.0	0.3	47.4	0.3	0.248
a^*^ value^1^	7.3	0.1	7.2	0.2	0.619
b^*^ value^1^	3.1	0.2	2.3	0.2	0.002
Intramuscular fat content (%)^1^	1.9	0.1	1.7	0.1	0.173
Muscle moisture content (%)^1^	74.1	0.1	74.3	0.1	0.188
Muscle protein content (%)^1^	23.1	0.1	23.2	0.1	0.802
Fat moisture content (%)^2^	7.2	0.5	9.7	0.5	0.001

L^*^ = 0 is completely black and L^*^ = 100 is completely white; positive a^*^ values mean red colors and negative a^*^ values mean green colors; positive b^*^ values mean yellow colors and negative b^*^ values mean blue colors.

1 Measurement done in Longissimus dorsi.

2 Measurement done in subcutaneous backfat.

*P* < 0.05 was used as significant differences between the groups.

The content of moisture in fat is related to the degree of unsaturation in fatty acids, and the method used here is rapid and cheap if many samples are analysed. In the current study, the fatty acid profile was also analysed, and this information provided similar information about the firmness of fat. There was a significant difference between Contr and RS pigs with respect to technological fat quality, and the RS pigs had 50% more PUFA and 2% more water in the fat.

### Sensory quality

From the sensory investigation of the eating quality of sausages, neck chops and bacon, we observed a good quality of the pig meat products. The bacon showed, however, an oily consistency. There were no other significant sensory differences between products made from the Contr and RS pigs for any of the products tested. An unchanged eating quality is in accordance to other studies in which vegetable ingredients were used to increase the omega-3 content of pig meat (Tikk et al. 2007; Beaulieu et al. 2009). In comparison, the use of marine feed ingredients as pig feed produces significant problems with the sensory meat quality (Øverland et al. 1996; Bryhni et al. 2002; Hallenstvedt 2011).

### Fatty acid analyses of feed, meat, and fat

The experimental diet RS was supplemented with RS, which resulted in a pig meat enriched in n3 LCPUFAs compared to regular pig meat. The RS meat contained approximately 44 mg EPA+DPA+DHA/100 g meat compared to around 36 mg/100 g meat in the control group (the meat in both treatment groups contained approximately 2% fat). This level of n3 LCPUFA was in accordance with the levels reported for pig meat (DTU 2009), and the increase in n3 LCPUFA in meat was approximately 20% in the RS group compared to controls. This increase in the muscle content of n3 LCPUFA following the supplementation of ALA to pigs was in agreement with other studies (Beaulieu et al. 2009; Bertol et al. 2013). The increase in n3 LCPUFA in backfat was roughly 40% in the RS group compared to the backfat in control pigs. The profile of the other fatty acids in the meat was similar in the RS and control group, with the exception of ALA being 2.4 times higher in the RS group. In the backfat, the concentration of ALA was three times higher in the RS group compared to the controls, and palmitic and stearic acid (16:0 and 18:0) were 20–30% lower in the RS group compared to the controls (Table6). The RS diet resulted in a pig meat with a favorably reduced ratio between the omega-6 and omega-3 fatty acids being 4.7:1, compared to 6.6:1 in the control group. The reduction in AA was approximately 10% in the RS compared to control meat, and the ratio AA/EPA was 4.5:1 in meat from the RS pigs, and 7.8:1 in the control meat (Table6). In the backfat, the ratios were similar, but even more pronounced: the AA/EPA ratio was 3.2:1 and the omega-6/omega-3 ratio was 4:1 in the RS pigs and the corresponding values in the controls were 6.5:1 and 8:1. This shows that it is possible to increase the omega-3 fatty acids and, notably, to reduce the ratios between the omega-6 and omega-3 fatty acids in both pig meat and lard by feeding the pigs a diet enriched in RS. The differences were highly significant. It is interesting to note that the response to diets on fatty acid composition was even larger in lard than in the meat.

**Table 6:** Fatty acid composition (g/100 g fat) and fatty acid indices in meat (M. longissimus dorsi) and subcutaneous fat from pigs fed either a control diet (Contr) (*n* = 15) or a diet supplemented with rapeseed products (RS) (*n* = 15). The mean, SEM and *t*-test (*P*) in the meat and backfat in the two treatment groups are shown

	Meat					Backfat				
	Control		RS			Control		RS		
	Mean	SEM	Mean	SEM	*P*	Mean	SEM	Mean	SEM	*P*
C14:0	1.12	0.054	1.02	0.045	0.171	1.31	0.036	1.14	0.024	0.001
C16:0	21.8	0.306	20.7	0.388	0.030	24.7	0.253	20.7	0.303	<0.001
C16:1n7	3.04	0.131	2.48	0.145	0.008	2.28	0.101	1.67	0.070	<0.001
C18:0	11.8	0.145	11.4	0.174	0.111	14.1	0.426	10.8	0.290	<0.001
C18:1c9	32.5	0.720	32.9	0.909	0.698	38.6	0.417	41.0	0.368	<0.001
C18:2n6 (LA)	11.8	0.529	13.1	0.684	0.153	10.1	0.389	13.6	0.496	<0.001
C18:3n6	0.17	0.013	0.13	0.009	0.017	0.02	0.001	0.03	0.002	0.034
C18:3n3 (ALA)	0.50	0.015	1.21	0.047	<0.001	1.03	0.041	3.06	0.094	<0.001
C20:2n6	0.30	0.009	0.35	0.013	0.007	0.59	0.014	0.69	0.011	<0.001
C20:3n6	0.57	0.031	0.52	0.032	0.303	0.09	0.004	0.09	0.004	0.935
C20:3n3	0.06	0.003	0.15	0.007	<0.001	0.16	0.006	0.37	0.007	<0.001
C20:4n6 (AA)	3.27	0.228	3.00	0.202	0.400	0.18	0.008	0.18	0.007	0.697
C20:5n3 (EPA)	0.43	0.034	0.67	0.041	<0.001	0.03	0.003	0.06	0.004	<0.001
C22:5n3 (DPA)	0.83	0.052	1.03	0.063	0.024	0.15	0.007	0.23	0.008	<0.001
C22:6n3 (DHA)	0.55	0.059	0.48	0.036	0.287	0.10	0.010	0.11	0.011	0.726
AA/EPA	7.77	0.369	4.47	0.097	<0.001	6.49	0.437	3.192	0.100	<0.001
n-6/n-3	6.60	0.144	4.73	0.056	<0.001	7.97	0.107	3.99	0.047	<0.001
16:1n7/16:0	0.14	0.004	0.12	0.006	0.009	0.09	0.004	0.08	0.004	0.055
18:1c9/18:0	2.76	0.073	2.90	0.110	0.294	2.77	0.102	3.85	0.124	<0.001
EPA/ALA	0.85	0.051	0.55	0.024	<0.001	0.03	0.002	0.02	0.001	<0.001
DPA/ALA	1.63	0.068	0.85	0.039	<0.001	0.14	0.004	0.08	0.002	<0.001
DHA/ALA	1.08	0.096	0.39	0.024	<0.001	0.10	0.008	0.03	0.003	<0.001
DPA/EPA	1.97	0.065	1.53	0.039	<0.001	5.23	0.231	3.98	0.134	<0.001
DHA/DPA	0.65	0.037	0.47	0.023	<0.001	0.66	0.041	0.45	0.034	0.001
AA/LA	0.28	0.010	0.23	0.006	0.001	0.02	0.001	0.01	0.000	<0.001
EPA+DPA+DHA/ALA	3.56	0.200	1.79	0.074	0.000	0.27	0.012	0.13	0.005	<0.001

n6/n3 = (18:2n6 + 20:4n6)/(18:3n3 + 20:5n3 + 22:5n3 + 22:6n3).

*P* < 0.05 was used as a significant difference between the groups.

The obtained 20% increase in EPA+DPA+DHA, being 77 mg in one portion (175 g) of the RS meat compared to 63 mg in the meat from controls, might not seem to be high since the EFSA suggestions are 250 and 450 mg n3 LCPUFA/day (EFSA 2009; EFSA 2012). However, all matters count, and this increase might be of significance to people who are not eating fish and seafood, and where meat is their sole source for long-chain omega-3 fatty acids. Too much saturated fat may not be advantageous, since in in vitro culture studies saturated fatty acids have been shown to activate inflammation processes (Poledne 2013). However, epidemiological studies are less consistent regarding the negative health effects of saturated fats (Poledne 2013; Chowdhury et al. 2014).

In brief, the RS pork meat in the present study is a good meat product for human consumption when considering the increase in EPA and DPA, the reduced ratio between omega-6 to omega-3 fatty acids and the high level of Se. Still, pig meat does not come close to rivaling the omega-3 content of fish. However, it is possible that an optimal feed mixture for producing healthier pork meat should contain even more ALA, perhaps by adding some linseed oil, thereby bringing the ratio between sum omega-6 and sum omega-3 fatty acids even lower. This would provide new challenges in feed handling and storage, and it could require storage in dark and airtight containers.

### Fatty acid product/precursor ratios

The present increase in n3 LCPUFAs may not reflect what is achievable. Estimates of the metabolism of the fatty acids in muscle and adipose tissues are indicated by fatty acid product/precursor ratios (Table6, lower panel). The ratio 16:1n7/16:0 and 18:1c9/18:0 are estimates of delta-9 desaturase. The ratios EPA/ALA, DPA/ALA, DHA/ALA, AA/LA, and EPA+DPA+DHA/ALA are estimates of a combined outcome of the conversion of ALA and LA to the n3 LCPUFAS and to AA (Miyazaki and Ntambi 2011). As shown in Table6, lower panel, there are significant differences in most of the product/precursor ratios in both meat and backfat between the two diet groups. The ALA concentration in the feed is double in the RS feed compared to the control feed, and LA is 17% lower in the RS feed compared to the control feed. The doubling of ALA in the feed resulted in an approximate doubling of ALA in the meat and backfat in the RS pigs compared to the controls. EPA and DPA also increased in muscle and backfat, but not to the same extent, and the indices EPA/ALA, DPA/ALA, and DHA/ALA were approximately halved in the RS pigs compared to control pigs. This may indicate that the doubling in ALA intake does not increase the EPA, DPA, and DHA content proportionally to the increase in ALA. However, the concomitant decreased intake of LA by the RS pigs does not result in decreased LA and AA in muscle and backfat. The supplementation of RS in the feed has resulted in large alterations in the metabolism of PUFAs in the pig, and the product/precursor ratios indicates possible improvements toward a higher synthesis of n3LCPUFA from ALA, and a reduced synthesis of AA.

### Selenium

The Se concentration in meat was 0.3 mg Se/kg muscle in both the control and RS groups, following a supplementation of 0.2 mg Se from an organic Se source (Sel-Plex Premix), and 0.2 mg Se from an inorganic Se source (Selenpremiks), which is in accordance with previous studies (Surai 2006). The selenium concentration in both groups of pigs was higher than the reported commercial pig meat, being 0.14 mg/kg in fresh loin (Mat på data 2014). A portion (175 g) of the meat from the present study would yield approximately 52 g of Se, which is the approximate recommended daily intake for men and women (60 g and 50 g/day, NNR 2012). Upper levels of Se intake in humans have been discussed, and a “no observed adverse effect level” (NOAEL) has been reported at 850 g/day (Nordic Nutrition Recommendations 2004), and in the Nordic Nutrition Recommendations 2012, estimated upper intake levels for daily chronic intakes (UL) for selenium were determined to be 300 g/day. The Se nutrient density in the average Norwegian diet has been determined to be 63 g/10 MJ, (not corrected for cooking losses) (NNR 2012), which may be sufficient for an adequate incorporation into selenoproteins that have a wide range of positive effects on health such as antioxidative and anti-inflammatory effects. However, some persons are below the optimal intake, and an additional selenium intake may benefit people with a low Se status (Rayman 2012).

## Conclusion

There was a small difference in growth rate in the RS pigs compared to the controls, but no differences in feed conversion ratio, lean meat percentage, carcass value, or margin per pig for the two groups. Furthermore, there were no differences in sensory traits, drip loss and color of the meat between the two meat groups. However, the backfat of the RS pigs was softer and easier to cut, and it contained 2% more water and approximately 50% more PUFAs compared to controls.

If pigs were given RS and selenium in the feed, pig meat and backfat would be healthier for human consumption compared to the pig products presently on the market. A feed based on RS resulted in pork meat and backfat having an omega-6/omega-3 ratio of approximately 4:1, while the corresponding ratio was 6.5–8:1 for the control group fed conventional soya-based feed. One portion (175 g) of pig meat from the RS group contained 77 mg EPA DPA and DHA compared to 63 mg in the control meat. The supplementation of RS resulted in decreased product/precursor ratios for elongation and desaturation of LC PUFAs from PUFAs. The product/precursor ratios indicates possibility of higher production of n3 LCPUFAs following higher ALA supplementation. Feeding strategies to increase the content of n3 LCPUFA even more, and reducing the AA in meat and backfat should be tested out.

The use of organic selenium in pig feed resulted in Se content of meat on 0.3 mg/kg. One portion of pork meat (175 g) contained 52 mg Se, which corresponds to the daily recommended intake.

Meat products with higher levels of ALA, EPA, DPA, DHA, and Se is a good approach for increasing the intake of very long-chain omega-3 fatty acids and Se, and for correcting the dietary balance between omega-6 and omega-3 fatty acids. Healthier meat is a broader strategy for increasing the omega-3 and Se intake of human populations rather than relying only on fish resources that are already overexploited, on Se and omega-3 pills or trying to alter people's food habits toward a higher fish consumption. In addition, the increased use of locally produced or short traveled oilseed products is a desire.
